# Feasibility and potential benefit of pre-procedural CMR imaging in patients with ischaemic cardiomyopathy undergoing cardiac resynchronisation therapy

**DOI:** 10.1007/s12471-019-01360-6

**Published:** 2020-01-17

**Authors:** W. A. Gathier, O. A. E. Salden, D. J. van Ginkel, W. M. van Everdingen, F. A. A. Mohamed Hoesein, M. J. M. Cramer, P. A. Doevendans, M. Meine, S. A. J. Chamuleau, F. J. van Slochteren

**Affiliations:** 1 Department of Cardiology, University Medical Centre Utrecht, Utrecht, The Netherlands; 2 Department of Radiology, University Medical Centre Utrecht, Utrecht, The Netherlands; 3 CART-Tech B.V., Utrecht, The Netherlands; 4 Regenerative Medicine Centre Utrecht, Utrecht, The Netherlands; 5 Central Military Hospital, Utrecht, The Netherlands; 6 Netherlands Heart Institute, Utrecht, The Netherlands

**Keywords:** Cardiac resynchronisation therapy, Magnetic resonance imaging, Treatment planning

## Abstract

**Aim:**

To determine the feasibility and potential benefit of a full cardiac magnetic resonance (CMR) work-up for assessing the location of scarred myocardium and the region of latest contraction (LCR) in patients with ischaemic cardiomyopathy (ICM) undergoing cardiac resynchronisation therapy (CRT).

**Methods:**

In 30 patients, scar identification and contraction timing analysis was retrospectively performed on CMR images. Fluoroscopic left ventricular (LV) lead positions were scored with respect to scar location, and when placed outside scar, with respect to the LCR. The association between the lead position with respect to scar, the LCR and echocardiographic LV end-systolic volume (LVESV) reduction was subsequently evaluated.

**Results:**

The CMR work-up was feasible in all but one patient, in whom image quality was poor. Scar and contraction timing data were succesfully displayed on 36-segment cardiac bullseye plots. Patients with leads placed outside scar had larger LVESV reduction (−21 ± 21%, *n* = 19) compared to patients with leads within scar (1 ± 25%, *n* = 11), yet total scar burden was higher in the latter group. There was a trend towards larger LVESV reduction in patients with leads in the scar-free LCR, compared to leads situated in scar-free segments but not in the LCR (−34 ± 14% vs −15 ± 21%, *p* = 0.06).

**Conclusions:**

The degree of reverse remodelling was larger in patients with leads situated in a scar-free LCR. In patients with leads situated within scar there was a neutral effect on reverse remodelling, which can be caused both by higher scar burden or lead position. These findings demonstrate the feasibility of a CMR work-up and potential benefit in ICM patients undergoing CRT.

## Whats new?

- Implementation of a full cardiac magnetic resonance imaging (CMR) work-up to determine tissue characteristics at the site of the left ventricular (LV) lead in patients with ischaemic cardiomyopathy undergoing CRT.
- Implementation of a 36-segment bullseye plot to accurately delineate scarred myocardium.
- Pacing from scar-free segments results in a larger degree of reverse remodelling. A trend was seen towards superior reverse remodelling in patients with LV leads placed in a scar-free, late-contracting region (LCR) compared to pacing in a scar-free segment outside a LCR.
- This study provides further support for a role of CMR in real-time, guided LV lead deployment.

## Introduction

Cardiac resynchronisation therapy (CRT) is an effective therapy for patients with chronic heart failure, impaired left ventricular (LV) ejection fraction and prolonged QRS duration [1]. Yet, 30–40% of patients do not benefit from the treatment [2]. Patients with ischaemic cardiomyopathy (ICM) derive less benefit from CRT, with 50% of patients displaying volumetric or clinical non-response [2–5]. Both a larger scar burden and pacing in or near an area with myocardial scar are associated with a suboptimal response to CRT [6–10]. On the other hand, echocardiographic studies suggested that pacing in the region of latest contraction (LCR) is associated with improved CRT response [11, 12]. In current clinical practice, the LV lead is implanted empirically at the basal lateral segment, where statistically the best response is obtained. For individual ICM patients, the location of myocardial scar represents an additional requirement for the location of the LV lead. Given the wide variation in scar distribution and scar burden, as well as a heterogeneity of electrical activation patterns, pre-procedural determination of the location of myocardial scar and mechanical delay may be of key importance in these patients. With advancements in implanting techniques, such as real-time image-guided LV lead delivery, the snare technique and multipoint pacing, tailor-made individualised therapy has become available to patients undergoing CRT [13–16]. These advances call for image post-processing techiques that can determine the location of myocardial scar and delayed contraction, so that these areas can either be avoided or targeted. Cardiac magnetic resonance imaging (CMR) has been suggested as a promising tool for this purpose. Late gadolinium enhancement (LGE) CMR is the gold standard for determining the location and transmurality of scar tissue. Furthermore, tissue tracking software packages, such as CMR feature tracking (FT), can be used to perform LV contraction timing analysis on standard CMR-CINE images [17, 18]. In the present study, we therefore investigated the feasibility and potential benefit of a CMR-based approach to identify scar location, scar transmurality, and LV contraction timing. Furthermore, we assessed the effect of tissue characteristics (e.g. scar and delayed contraction) at the LV pacing electrode on LV reverse remodelling after CRT.

## Materials and methods

### Patient selection

Patients with ICM that had undergone CRT implantation and had a pre-implantation CMR scan and pre- and post-implant echocardiography acquisition were retrospectively included in the study. Patients received a CRT device in accordance with the ESC guidelines between 2006 and 2016 at the University Medical Centre Utrecht [19]. Standard CRT device implantation was performed with the LV lead placed empirically in a coronary vein overlying the LV free wall, the right atrial lead in the right atrial appendage and the right ventricular lead in the right apicoseptal segment. Patient medical records were screened for an ischaemic origin of heart failure based on the presence of ischaemic delayed enhancement on CMR-LGE sequences, prior coronary artery bypass grafting, or percutaneous coronary intervention. The study was approved by the local medical ethical committee (METC), by whom the need for informed consent was waived.

### Study design

**Fig. 1:**
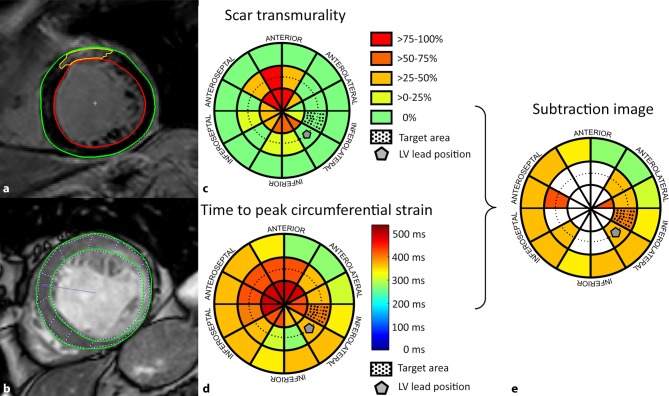
**a**–**e** Cardiac magnetic resonance (CMR) processing. **a** Segmentation of late gadolinium enhancement CMR sequences used to determine myocardial scar transmurality and position. **b** Feature tracking of CMR-CINE sequences used for strain analysis and determination of the area of latest time-to-peak circumferential strain. The *arrow* depicts the right ventricle hinge point. **c**, **d** 36-segment cardiac bullseye plots depicting segmental scar transmurality (**c**) and time-to-peak circumferential strain (**d**). The subtraction image (**e**) shows the contraction timing as shown in **d** with subtraction of scarred segments from (**c**). The left ventricular target area is depicted as a *dotted* segment and the left ventricular lead position is marked by a *pentagon*. *LV* left ventricular

**Fig. 2:**
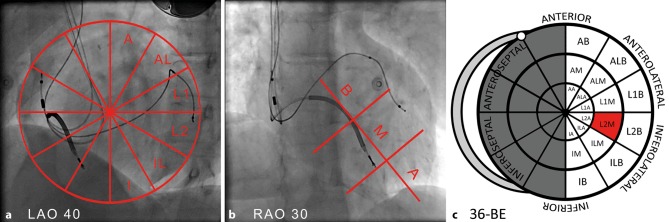
**a**–**c** Identification of the left ventricular lead position on fluoroscopy images. **a** Left anterior oblique (*LAO*) 40º view of the heart with the lateral part of the left ventricle divided into six segments (*A* anterior, *AL* anterolateral, *L1* lateral 1, *L2* lateral 2, *IL* inferolateral, *I* inferior) with a grid placed over the fluoroscopy image to determine the segment of pacing. **b** Right anterior oblique (*RAO*) 30º view of the heart divided in three levels (*B* basal, *M* mid, *A* apical). **c** 36-segment cardiac bullseye (*BE*) plot representing the fusion of the LAO 40º view and RAO 30º view. The distal pacing electrode, which was configurated for biventricular pacing, is located in the *red* segment

In all patients, the location of myocardial scar, the LCR and the location of the LV pacing electrode were determined and scored on cardiac bullseye plot models. This was done by dividing the LV myocardium into a custom-made 36-segment bullseye plot representation (Figs. 1 and 2). Subsequently, the association of the LV lead position with respect to the CMR-defined location of scar, LCR and its relation to LV reverse remodelling was assessed. LV reverse remodelling was evaluated in terms of LV end-systolic volume (LVESV) reduction.

### LV lead position

Assessment of the position of the programmed LV pacing electrode on fluoroscopic projections made during CRT implantation was performed by two investigators blinded to all other study data (interobserver variability κ = 0.92). The 30º right anterior oblique (RAO) view was used to determine the long axis position of the LV lead (basal, mid or apical), and the 40º left anterior oblique (LAO) view was used to determine the circumferential position of the LV lead on the free wall (anterior, anterolateral, lateral 1, lateral 2, inferolateral and inferior) (Fig. 2).

### CMR analysis

CMR scans were performed on a 1.5T MRI scanner (Achieva, Philips Medical Systems, Best, The Netherlands) using a standardised protocol as described in detail previously [14]. Scar segmentations were processed using Segment CMR software (Medviso, Lund, Sweden). With this approach, scar transmurality per myocardial segment was evaluated in each patient, as well as total LV scar burden (Fig. 1). Scar-free segments were defined as segments with scar transmurality of 0–5%. This was done to correct for artefacts and noise from, for instance, blood pool or epicardial fat. For detection of the segments of latest mechanical contraction, time to peak (TTP) analysis was performed on short-axis CMR-CINE images using CMR-FT software (TomTec Arena, 2D Cardiac Performance Analysis MR, Unterschleißheim, Germany) as described previously [14]. In short: endo- and epicardial borders of the short-axis CMR-CINE sequences were drawn manually in the end-diastolic frame for all slices. CMR-FT software then automatically followed the myocardial borders throughout the remainder of the cardiac cycle. This resulted in automatically generated circumferential strain data, which were manually checked and corrected when necessary to ensure optimal strain data. Scar transmurality and TTP-strain data were expressed on cardiac bullseye plots using an in-house-developed software program running in MATLAB and Statistics Toolbox (The MathWorks, Inc., Natick, MA, USA) (Fig. 1). The location of the fluoroscopic LV pacing electrode was scored in a blinded fashion as within an area of scar (‘within scar’) or at a scar-free site (‘outside scar’). In patients with an LV lead in a scar-free segment, the LV lead location was subsequently scored with respect to the segment with the highest TTP strain (latest contracting region) and defined as ‘within the LCR’ or ‘outside of the LCR’.

### Statistics

Statistical analysis was performed using IBM SPSS Statistics 25 software (IBM, Armonk, NY, USA). Continuous variables were tested for normality with a Shapiro-Wilk test, and were described using mean ± standard deviation or, in the case of non-normal distribution, with the median (interquartile range). Categorical data were described by an absolute number of occurrences and associated frequency (%). Between-group comparisons were performed with Mann-Whitney U tests (continuous data with non-normal distribution), unpaired Student *t*-test (normally distributed data) and Pearson chi-square test or, if there was an expected cell count of <5, Fisher’s exact test (dichotomous variables). A *p*-value of <0.05 was considered to be significant and all tests were two-tailed.

## Results

### Baseline characteristics

A total of 35 patients met all the inclusion criteria. In four patients echocardiography quality was insufficient. CMR processing was feasible in all but one patient, in whom CMR quality was insufficient to perform FT analysis. Therefore, 30 patients were included in the analysis; their baseline characteristics are described in Tab. 1.

**Table 1:** Baseline characteristics

	All patients	LV lead in scar-free region and LCR	LV lead in scar-free region, not in LCR	LV lead within scar
Patient characteristics	**(*n*** **=30)**	**(*n*** **=6)**	**(*n*** **=13)**	**(*n*** **=11)**
Age at implantation (years)	69.9 ± 5.8	68 ± 7	72 ± 5	68 ± 6
Male gender, *n* (%)	24 (80)	4 (66.7)	10 (76.9)	10 (90.9)
LBBB conduction, *n* (%)	23 (76.7)	5 (83.3)	10 (76.9)	8 (72.7)
QRS duration (ms)	150 ± 19	151 ± 29	146 ± 18	154 ± 14
NYHA, *n* (%)				
I/II	13 (43.3)	2 (33.3)	6 (46.2)	5 (45)
III/IV	15 (50)	3 (50)	7 (53.8)	5 (45)
Scar burden, % (interquartile range)	19 (14–24)	13 (5–31)	14 (12–22)*	21 (18–44)*
LV end-systolic volume (ml)	151 ± 56	174 ± 79	143 ± 39 (*p* = 0.06)	148 ± 62
LV end-diastolic volume (ml)	198 ± 63	215 ± 91	193 ± 41	196 ± 72
LV ejection fraction (%)	24.8 ± 7.0	20 ± 5	26 ± 7	26 ± 7*
Comorbidities, *n* (%)	10 (33.3)			
Atrial fibrillation	9 (30)	1 (16.7)	5 (38.5)	3 (27.3)
Hypertension	16 (53.3)	5 (83.3)	8 (61.5)*	3 (27.3)*
Smoking	19 (63.3)	5 (83.3)	7 (53.8)	7 (63.6)
Medication, *n* (%)				
Beta blocker	21 (70)	4 (66.7)	11 (84.6)	6 (54.5)
ACE-i/ARB	29 (96.7)	5 (100)	14 (100)	10 (90.9)
Diuretics	25 (83.3)	5 (83.3)	12 (92.3)	8 (72.7)

Data presented as mean with standard deviation, median with interquartile range

*LBBB* left bundle branch block according to ESC 2013 criteria [2]; *NYHA class* New York Heart Association functional classification; *LV* left ventricular; *ACE‑i* angiotensin-converting enzyme inhibitor; *ARB* angiotensin receptor blocker

* Significant difference (*p* < 0.05)

### LV lead position in relation to myocardial scar, delayed contraction and LVESV reduction

**Fig. 3:**
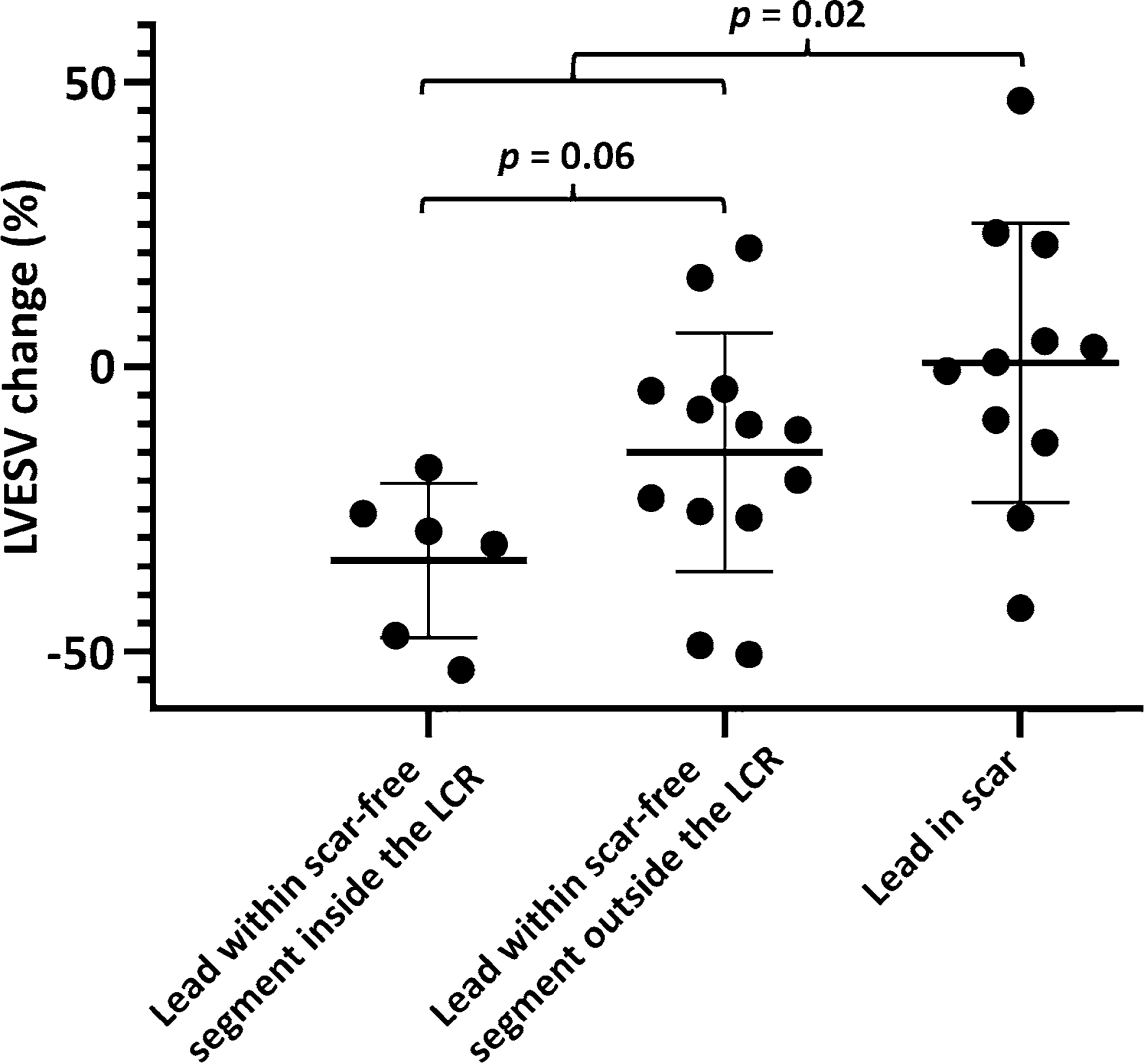
Echocardiographic response versus lead location in relation to the myocardial scar and the region of latest contraction (*LCR*). Relation between scar location, the location of the LCR, the LV pacing electrode and its relation to left ventricular end systolic volume (*LVESV*) change

Eleven patients (37%) had LV leads situated in scarred myocardium. Of these, three patients had the LV lead placed in a segment with >75% scar transmurality, two in an area with >50–75% scar transmurality, four in an area with >25–50% scar transmurality and two in an area with >0–25% scar transmurality. Patients in whom the LV lead was placed in a scar-free segment (*n* = 19) had a significantly greater LVESV reduction at follow-up compared to patients in whom the LV lead was placed within scar (−21 ± 21% vs 1 ± 25% respectively, *p* = 0.02). There was a trend towards larger LVESV reduction in patients with LV leads placed in a scar-free segment as well as in the LCR compared to patients with leads in a scar-free segment but not in the LCR (−34 ± 14% vs −15 ± 21%, *p* = 0.06) (Fig. 3).

### Myocardial scar burden

The amount of reverse remodelling after CRT is affected not only by the presence or absence of myocardial scar or significant mechanical delay at the LV pacing electrode, but also by the total LV scar burden. Median LV scar burden was 19% (14–24). Scar burden was significantly higher in patients with leads within scar compared to patients with leads in scar-free myocardial segments [21% (18–44) vs 15% (10–22), *p* = 0.02]. Scar burden did not vary between patients with leads in a scar-free segment as well as in the LCR and patients with leads in a scar-free segment but outside of the LCR [15% (5–35) vs 15% (11–22), *p* = 0.677].

## Discussion

The present study demonstrates the feasibility and potential benefit of a CMR work-up for the assessment of the optimal site for LV pacing based on myocardial scar transmurality and the area of latest contraction. For the visualisation of scar and delayed contraction, 36-segment cardiac bullseye plots were used. LVESV reduction was most evident in patients with LV leads situated in scar-free segments. In addition, there was a trend towards improved LVESV reduction when the LV lead was placed outside of scar and in the LCR compared to outside of scar and outside the LCR. Because scar burden did not differ significantly between these groups, it is possible that this trend is based on a favourable lead position in these patients. In patients with LV leads placed within scar there was an overall neutral effect on reverse remodelling. This can be caused by both higher scar burden or the LV lead position. These results are in line with those of previous publications that show that pacing within scar is associated with CRT non-response, while pacing outside of scar and in the area with most delayed contraction leads to superior results [9–12, 20].

### Pre-procedural identification of target sites for LV lead delivery

Despite the promising results from echocardiographic studies showing the benefit of targeted LV lead placement, such as the TARGET and STARTER trials, no prospective, randomised clinical studies currently have been published using CMR to guide LV lead delivery in CRT [11, 12]. An important limitation of echocardiography is the inability to visualise scar. Yet, in ICM patients undergoing CRT, scar identification is of key importance given the association between scar and poor outcomes [6–10]. CMR, in contrast to echocardiography, is a useful imaging modality to identify and quantify both scar and dyssynchrony in a three-dimensional fashion and, therefore, may represent the optimal imaging modality for treatment planning [20, 21]. In line with our results, Taylor et al. demonstrated in a retrospective study that an LV lead position over non-scarred, late-contracting segments, assessed with CMR, was associated with improved echocardiographic response and superior outcomes in CRT patients [20]. In contrast to the present study, only 50% of patients had ICM. Importantly, Taylor et al. did not assess total LV scar burden and its impact on the relation between the LV lead location and CRT response [20]. In addition, a novelty of the present study is that we used smaller LV segments to visualise scar and mechanical delay. When displaying these data on American Heart Association 17-segment cardiac bullseye plots, we believe that the accuracy of CMR is not fully exploited; hence we created smaller LV segments.

### Clinical implications

This study shows that CMR is a potentially useful tool that could be used to identify target sites for LV stimulation and, hence, prospectively plan and guide LV lead delivery in patients undergoing CRT implantation. This is especially valuable in patients with scarred myocardium, in whom it is unlikely that a single, empirical location for LV lead placement will adequately resynchronise all patients. Eleven patients (37%) in our study had LV leads positioned in scarred myocardium while a pre-implantation CMR-LGE scan was at the implanting cardiologist’s disposal. These data suggest that pre-procedural visual inspection of the plain MRI dataset can not always prevent lead implantation in scarred segments. More advanced, image-guided LV lead implantation, in which scarred myocardium or target sites for LV lead delivery are projected on top of the live fluoroscopy during implantation, might overcome this problem [13, 14, 21]. When retrospectively assessing the availability of a suitable coronary branch on fluoroscopy in the current study, a suitable alternative target vein was available in a scar-free segment in nine out of these 11 patients. This is an interesting finding because it further fuels the concept of careful assessment of CMR images before performing CRT implantation. Still, we recognise that we do not know whether acceptable capture thresholds without phrenic nerve stimulation would have been available in non-scarred segments in these nine patients.

### Limitations and challenges

The two main limitations of this study are the retrospective design and small sample size. This is caused by the fact that we included only patients with myocardial scar on CMR-LGE scans that were performed before CRT implantation. Differences in baseline characteristics between patients could have influenced our results. For example, patients with LV leads placed within scar had more unfavourable characteristics at baseline (e.g. higher scar burden, lower frequency of left bundle branch block and more males). The advantage of CMR-FT is that it is a relatively easy technique for contraction timing analysis since it can be performed on CMR-CINE images, which are obtained during standard cardiac imaging protocols. Still, there are some limitations when assessing TTP-strain data. Both electrical substrates (which are generally responsive to CRT) and non-electrical substrates, such as hypocontractility and myocardial scar (which do not respond to CRT) may cause TTP-strain delay [22]. To avoid noise from scarred segments causing TTP-strain delay, we determined contracting timing only in segments outside scarred myocardium (Fig. 1e). Due to between-group differences and the restrospective study design we cannot draw firm conclusions regarding the superior effect of placing the LV lead in a CMR-defined LV target segment. Yet, the present study is a feasibility study of a full-CMR work-up. Larger trials are needed to further determine whether this approach leads to improved CRT response.

## Conclusion

This study demonstrates the feasibility and potential benefit of a CMR work-up to determine optimal LV pacing sites in ICM patients undergoing CRT implantation. Patients in whom the LV lead was placed in a scar-free region with most delayed contraction showed marked LV reverse remodelling, while in patients with leads in scarred segments there was an overall neutral effect on LVESV change.
